# Simultaneous Detection of Food Contaminants Using Surface-Enhanced Raman Scattering (SERS): A Review

**DOI:** 10.3390/foods14172982

**Published:** 2025-08-26

**Authors:** Lixin Ma, Ruiyun Zhou, Limei Yin, Li Sun, En Han, Junwen Bai, Jianrong Cai

**Affiliations:** 1 School of Food and Biological Engineering, Jiangsu University, Zhenjiang 212013, China; lixinma99@163.com (L.M.);; 2 School of Agricultural Engineering, Jiangsu University, Zhenjiang 212013, China

**Keywords:** surface-enhanced Raman scattering, simultaneous detection, Raman reporter molecules, food contaminants

## Abstract

Surface-enhanced Raman scattering (SERS) technology has garnered significant attention for the detection of trace food contaminants, due to its exceptional sensitivity, non-destructive nature, and molecular fingerprinting capabilities. Currently, SERS applications in the simultaneous detection of multiple contaminants have advanced rapidly. SERS-based simultaneous detection strategies are generally categorized into label-free and labeled detection methods. Labeled detection can be further divided into SERS encoding detection and spatial isolation detection, with Raman reporter molecules playing a key role in SERS encoding. This article reviews the strategies, principles, common Raman reporter molecules, and practical applications of SERS-based simultaneous detection. Additionally, this article explores the challenges and future directions of SERS technology in contaminant detection, with an emphasis on the development of stable, intelligent substrates; improvements in analytical algorithms; and the creation of portable, on-site detection platforms. This study seeks to offer valuable insights into the development of SERS-based methods for simultaneously detecting multi-contaminants.

## 1. Introduction

Food safety is a critical issue in global public health, directly related to human health and social and economic development [1]. Food contaminants are one of the main factors causing foodborne diseases [2]. Common food contaminants include harmful microbes, mycotoxins, pesticide residues, antibiotic residues, and illegal additives [3,4,5,6,7]. These contaminants will remain in the environment or food and accumulate in human tissues through the food chain, potentially causing acute poisoning, chronic diseases, and even cancer [8,9]. Additionally, pollutants produced during food processing, such as polycyclic aromatic hydrocarbons, and environmental pollutants, like polychlorinated biphenyls, further exacerbate food safety risks [10,11]. Therefore, establishing rapid, sensitive, and accurate detection technologies is of vital importance to ensuring food safety.

Although traditional detection methods such as mass spectrometry, gas chromatography, and high-performance liquid chromatography (HPLC) offer broad applicability and high accuracy, they have limitations including complex operation, expensive equipment, and insufficient multi-target detection capabilities [12]. Currently, numerous neoteric methods are employed to detect food hazardous contaminants, such as fluorescence methods [13,14], enzyme-linked immunosorbent assay [15,16], colorimetry [17,18], isothermal amplification methods [19], and electrochemical techniques [20,21]. Although these methods demonstrate high sensitivity, they present certain limitations: fluorescence techniques are susceptible to photobleaching and require expensive probes; electrochemical methods require improved stability; and isothermal amplification necessitates nucleic acid extraction, making it vulnerable to matrix interference. Therefore, it is essential to develop simple and rapid detection methods.

Raman spectroscopy, a molecular vibrational spectroscopy technique, offers several advantages including non-destructive analysis, rapid measurement, and insensitivity to moisture interference. While this technique provides valuable information about molecular chemical structures, it suffers from inherently weak signals. Surface-enhanced Raman scattering (SERS) technology is a promising analytical technique that has evolved from traditional Raman spectroscopy. It utilizes noble metal nanoparticles as the substrate to significantly enhance the Raman signal of the target analyte, effectively overcoming these limitations and being particularly suitable for the sensitive detection of trace substances, providing a unique solution for achieving rapid and highly sensitive detection [22,23]. This enhancement occurs when target molecules are adsorbed on or near the surfaces of metallic nanostructures, allowing detection sensitivity to reach the single-molecule level [24]. The enhancement mechanisms of SERS technology can be mainly classified into electromagnetic enhancement based on electromagnetic fields and chemical enhancement based on charge transfer [25]. The local surface plasmon resonance model is a popular topic in the research of electromagnetic enhancement mechanisms [26]. It indicates that when the incident laser shines on the rough surface of a noble metal, the free-moving electrons on the noble metal surface will generate plasmon resonance, thereby increasing the electromagnetic field on the metal nano-surface. Given that localized surface plasmon resonance amplifies both the incident and scattered light, even a modest enhancement of the local electromagnetic field can result in a substantial increase in the SERS signal. The electromagnetic field enhancement effect is not selective and has the same enhancement effect for all substances. The chemical enhancement mainly lies in the charge transfer between the SERS enhancement substrate and the molecules adsorbed on its surface, which greatly increases the polarization rate of the adsorbed molecules, and the chemical properties of the molecules themselves also have a significant impact on the enhancement effect. SERS technology has the advantages of being rapid, non-destructive, highly sensitive, selective, and having low requirements for sample preparation. SRES has found broad applications across diverse fields, including environmental monitoring [27,28], early disease diagnosis [29,30], material science [31,32], biological imaging [33], and food safety [34,35].

SERS technology has been widely used for the single detection of food contaminants. For instance, Chen et al. developed an effective and sensitive SERS substrate for the rapid detection of thiabendazole (TBZ) residues in fruit samples. The limits of detection (LODs) in apple juice and peach juice were 0.032 and 0.034 mg/L, respectively [36]. Barimah et al. proposed a silver nanosensor labeled with 3-aminobenzeneboronic acid and combined it with chemometric algorithms to achieve the prediction of total arsenic content in tea leaves [37]. SERS technology is characterized by sharp peaks and exceptionally narrow peak widths, which significantly reduce peak overlap and enable the simultaneous detection of multiple targets [38]. Additionally, compared with the detection of a single substance, simultaneous detection has multiple advantages, as it can reduce the analysis time and cost and also provide a larger sample output per unit and higher analysis efficiency [39]. Therefore, researchers have shown a strong interest in studying multiple pollutants simultaneously and have made significant progress in detecting bacteria, mycotoxins, pesticides, and antibiotics.

**Figure 1:**
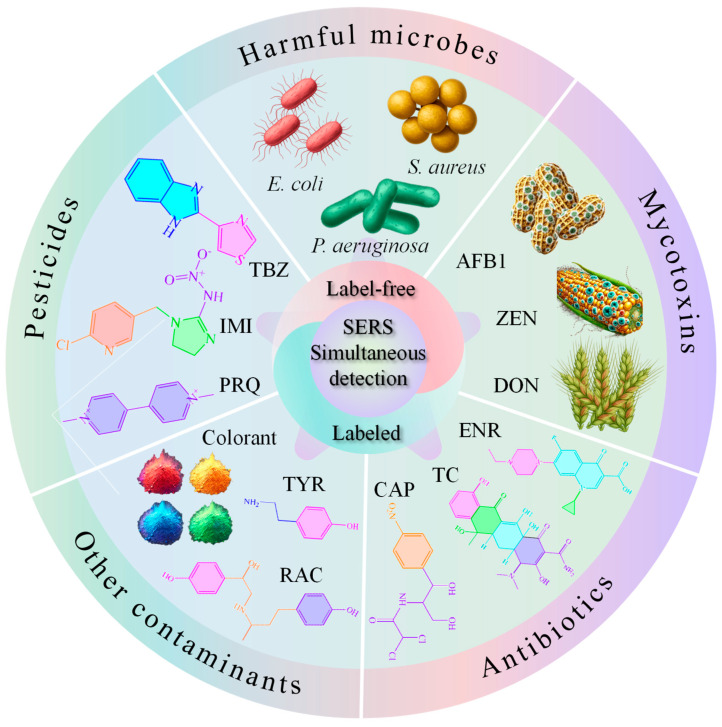
Schematic diagram of SERS technology for simultaneous detection of food contaminants. *S. aureus*, *Staphylococcus aureus*; *E. coli*, *Escherichia coli*; *P. aeruginosa*, *Pseudomonas aeruginosa*; ENR, enrofloxacin; TC, tetracycline; CAP, chloramphenicol; IMI, imidacloprid; TBZ, thiabendazole; PRQ, paraquat; TYR, tyramine; RAC, ractopamine; AFB1, aflatoxin B1; ZEN, zearalenone; DON, deoxynivalenol.

Considering that the application of SERS technology in food safety detection has been widely studied, several reviews have emerged, each focusing on distinct aspects. However, these reviews typically either concentrate on the detection of specific substances or predominantly address the synthesis of particular enhanced substrates [40,41,42]. Moreover, researchers generally concentrate on single-component detection or the individual detection of multiple components in food contaminants using SERS technology, without emphasizing the simultaneous detection of multiple contaminants [43,44]. In real-world scenarios, food samples are often exposed to a variety of contaminants simultaneously. Therefore, this paper aims to underscore the importance of SERS simultaneous detection technology in food safety by systematically summarizing the development of SERS-enhanced substrates, the use of Raman tag molecules, and their application in contaminant detection (Figure 1). Additionally, this paper will analyze the challenges and future prospects of this technology.

## 2. SERS Simultaneous Detection Strategy

SERS simultaneous detection technology is typically employed in food safety testing using two strategies: label-free detection and labeled detection. Label-free detection utilizes SERS substrates to directly enhance the signals of target molecules, thereby obtaining the Raman fingerprint spectra of the target substances, identifying their characteristic peaks, and achieving qualitative and quantitative analysis based on the positions and intensities of these peaks [45,46]. Compared with label-free detection, labeled detection is an indirect method primarily aimed at ions and molecules with relatively small scattering cross-sections. Sometimes, it is necessary to combine algorithms such as chemometrics and machine learning to improve the sensitivity and accuracy of detection [47]. The performance of this direct detection method is mainly related to the enhancement effect of the substrate and the Raman scattering cross-section of the target object itself. Therefore, this method is not suitable for the detection of target substances with extremely small Raman scattering cross-sections, as the Raman characteristic peaks of the target substances may not be obtained. The Raman scattering cross-sectional characteristics of the target object itself are difficult to change. Therefore, developing new SERS substrate materials with good enhancement effects is conducive to expanding the application scope of this method. At present, researchers have developed many new types of substrate materials, such as Au nano-dumbbells [48], nanorods [49], nanoflowers [50], nanotriangles [51], nanostars [52], ZnO@ZIF-8 [53], WO_3−x_ nanowire/WSe_2_ heterostructures [54], and chiral CNT/TiO_2_ hybrids [55].

Labeled detection provides Raman signals by Raman reporter molecules. The signal intensity reflects the concentration of the target substance, enabling sensitive detection of the target. Usually, labeled detection also requires the addition of molecular recognition elements to achieve target capture, with specificity [56]. Simultaneous labeled detection can be further summarized into two methods according to the actual sensing scheme: spatial separation detection and SERS coding detection. Spatial separation detection refers to the process of dividing the detection area during detection, detecting one target substance in each area, and collecting Raman signals from different areas to obtain quantitative results of multiple substances. This sensing method is usually combined with lateral flow test strips, with each T-line, respectively, coupled to the molecular recognition elements (antibodies, antigens, aptamers, cDNA) of their respective target substances [57]. In spatial separation detection, the Raman reporter molecules used by different signal probes can be the same type of Raman reporter molecules or different ones, because the required Raman signals have been confirmed spatially and no additional specific Raman signal encoding is needed. However, SERS encoded detection must encode the Raman signal in order to detect multiple substances in the same area [58]. This method usually employs multiple Raman reporter molecules, whose Raman characteristic peaks can be well distinguished [59].

**Figure 2:**
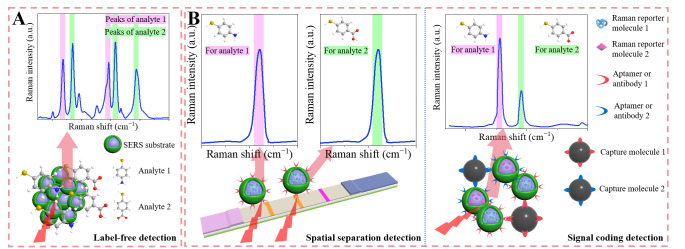
Illustration of SERS simultaneous detection strategies, including (**A**) label-free detection and (**B**) labeled detection.

Generally, when the target substance exhibits a strong SERS response, its Raman spectral data can be directly obtained for label-free detection. Conversely, when the SERS response of the target substance is weak, labeled detection is typically used, using Raman label molecules to reflect analytes’ concentration. For example, pesticides such as thiram (TRM) and TBZ can be quantitatively analyzed more effectively through direct detection [60,61]. However, for most mycotoxins, such as zearalenone (ZEN) and aflatoxin, labeled detection is necessary for accurate quantification [62]. Figure 2 shows the common strategies for SERS simultaneous detection, including label-free detection, spatial separation detection, and SERS coding detection.

Relatively speaking, the spatial separation detection in labeled detection and label-free detection is relatively simple when constructing SERS substrates or SERS signal probes. SERS signal coding detection requires the selection of Raman reporter molecules with non-interfering Raman characteristic peaks. Therefore, understanding common Raman reporter molecules is of great significance for the research of SERS coding detection, and this paper introduces it as a key point.

**Figure 3:**
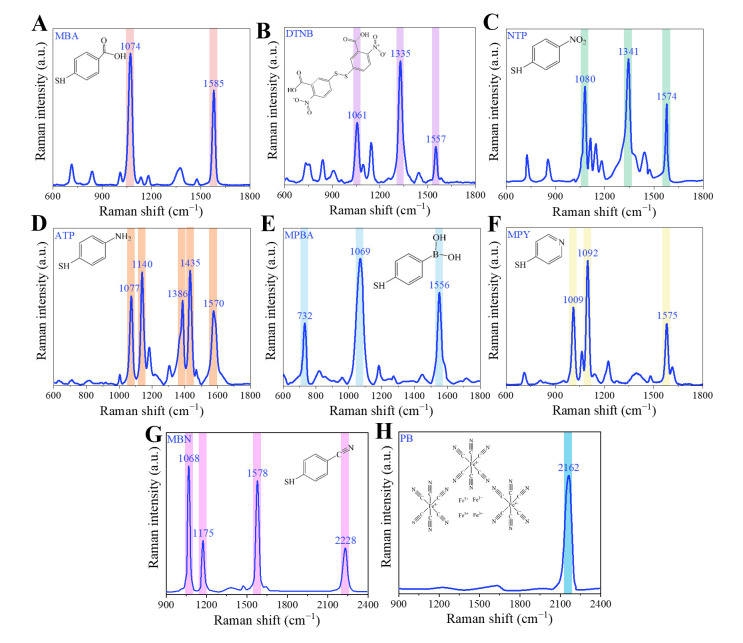
Raman spectra of different Raman reporter molecules: (**A**) MBA, (**B**) DTNB, (**C**) NTP, (**D**) ATP, (**E**) MPBA, (**F**) MPY, (**G**) MBN, (**H**) PB. The SERS spectra were collected using the Confocal Micro-Raman imaging spectrometer (XploRA PLUS, HORIBA, Paris, France) with an incident laser at 638 nm (laser intensity of 50 mW, integration time of 2 s).

The most common Raman reporter molecules used for SERS coding detection are 4-mercaptobenzoic acid (MBA), 5,5′-dithiobis-(2-nitrobenzoic acid) (DTNB), 4-nitrothiophenol (NTP), 4-aminothiophenol (ATP), 4-mercaptophenylboronic acid (MPBA), 4-mercaptopyridine (MPY), 4-(mercaptomethyl) benzonitrile (MBN), and Prussian blue (PB). Due to the differences in structure, the positions of their characteristic peaks are also different (Figure 3). Furthermore, due to the different orientations of the Raman signal molecules on various SERS substrates, the positions of the Raman characteristic peaks will undergo a certain degree of shift [63].

(1) MBA

The structure of MBA is that the hydrogen atoms on the first and fourth carbon atoms of the benzene ring are, respectively, replaced by a carboxyl group (–COOH) and a sulfhydryl group (–SH). Under acidic conditions, the –COOH of the MBA remains protonated, whereas under basic conditions, it deprotonates to form –COO^−^, altering the electron density of the aromatic ring. Additionally, electrostatic repulsion between the deprotonated molecules decreases their adsorption onto the SERS substrate [64]. Consequently, the pH value influences both the position and intensity of Raman signal peaks of MBA. MBA contains –SH, it can be combined with Au and Ag nanoparticles through Au–S bonds and Ag–S bonds. The Raman characteristic peaks of MBA are at 1074, 1386, and 1585 cm^−1^ [65]. Among them, the Raman characteristic peaks at 1585 and 1074 cm^−1^, which belong to ring breathing mode and axial ring deformation mode, are very strong and are usually used as quantitative indicator peaks of the target substance [66].

(2) DTNB

5,5′-Dithiobis (2-nitrobenzoic acid), abbreviated as DTNB, is also known as Ellman reagent. It is commonly used for the detection of –SH. DTNB is composed of two symmetrical 2-nitrobenzoic acid groups connected by a disulfide bond (–S–S–). Due to its relatively high Raman characteristic peak intensity, it is also frequently used as a Raman reporter molecule. DTNB has obvious characteristic peaks at 1061 cm^−1^, 1335 cm^−1^, and 1557 cm^−1^, which contribute to C–N bending, C–N stretching mode, and symmetric nitro group (–NO_2_) stretching mode, respectively. Among these, the 1335 cm^−1^ peak is usually the strongest Raman characteristic peak and is often used as the quantitative peak [67].

(3) NTP

The molecular formula of the NTP molecule is C_6_H_5_NO_2_S. The first carbon and fourth carbon of the benzene ring are, respectively, connected to –SH and –NO_2_. Similar to MBA, NTP can form strong covalent bonds with the metal surface through S atoms, thereby stably adsorbing on the SERS active substrate. The Raman characteristic peaks of the NTP molecule are located at 1080, 1341, and 1574 cm^−1^. The intensity at 1341 cm^−1^ is the highest and is usually selected as the quantitative peak [68].

(4) ATP

The ATP molecule is a benzene ring derivative formed by replacing the fourth and first carbon atom of the benzene ring with amino (–NH_2_) and –SH. Under acidic conditions, the –NH_2_ is protonated to –NH_3_^+^, which may enhance electrostatic adsorption [69]. In contrast, under alkaline conditions, the –SH deprotonates to form –S^−^, potentially leading to the formation of a more stable Ag–S or Au–S bond. Therefore, the protonation states of the –NH_2_ and –SH groups of ATP influence the interaction between the molecule and the metal surface, which may alter the posture of ATP on the SERS substrate, resulting in a slight shift in the Raman peak. –NH_2_ is a polar group, which can combine with other molecules (such as biological molecules, probe molecules) through hydrogen bonds or electrostatic interactions and can also achieve targeted recognition of target substances through –NH_2_ modification (facilitating the connection of aptamers and antibodies). Generally, the characteristic peaks of ATP molecules are located at 1004, 1077, 1140, 1195, 1386, 1435, and 1570 cm^−1^ [70]. Among them, the Raman characteristic peaks at 1140 and 1435 cm^−1^ are relatively strong.

(5) MPBA

The chemical formula of the MPBA molecule is C_6_H_7_BO_2_S. The first carbon atom in the benzene ring and the fourth carbon atom are, respectively, connected to the –SH and the boronic acid group. The –SH can form strong covalent bonds with the metal surface through the sulfur atom, thereby stably adsorbing on the SERS active substrate. The boronic acid group, as a “functional group”, possesses unique chemical reactivity and can specifically bind to molecules with cis diol structures (such as sugars, nucleotides, dopamine) [71]. This is the core advantage that distinguishes it from other SERS labels. The boronic acid group in MPBA can bind to the peptidoglycan in the bacterial cell wall, thereby capturing various bacteria [72]. The Raman characteristic peaks of the MPBA molecule are located at 732, 1070, 1185, and 1556 cm^−1^ [73]. Among them, the intensity at 1070 cm^−1^ is the highest, and it is usually selected as the quantitative peak.

(6) MPY

MPY contains a –SH on the fourth carbon atom and can be adsorbed on the substrate surface by forming Au–S bonds and Ag–S bonds with Au and Ag. The nitrogen atom (N) on the pyridine ring has lone pairs of electrons, which gives it certain basicity (ability to accept protons) and polarity. It can combine with other molecules through hydrogen bonds, electrostatic interactions, or coordination bonds. MPY has characteristic peaks near 712, 1008, 1092, 1212, 1575, and 1610 cm^−1^. The coupling peak of the annular breathing vibration and the stretching vibration in the C–S plane at 1092 cm^−1^ is the strongest peak [74].

(7) MBN

The MBN molecule contains a benzene ring. At the first position of the benzene ring, there is –SH, and at the fourth position, there is a cyano group (–C≡N). MBN is adsorbed onto the surfaces of SERS-active substrates such as Au and Ag through –SH, forming an ordered monolayer film. The characteristic Raman peaks of–C≡N are located in the biological silent region (1800 cm^−1^ to 2800 cm^−1^). MBN observed significant Raman characteristic peaks in the “fingerprint” region (1070, 1175 and 1579 cm^−1^) as well as in the “biological silence” region (2228 cm^−1^) [75]. Among them, the Raman peak at 2228 cm^−1^ is usually used for anti-optical interference detection or as an internal standard peak for ratio Raman spectroscopy [76].

(8) PB

PB is a ferrocyanide complex. Fe^2+^, Fe^3+^, and –C≡N form a three-dimensional grid structure through coordination bonds. The –C≡N group in the PB molecule is the key Raman active unit. Its C≡N stretching vibration peak (approximately 2156 cm^−1^) has high intensity and a sharp peak shape, and it is located in the region where the Raman signal of biological samples (such as proteins, nucleic acids) is extremely weak [77]. This can effectively avoid matrix interference and is suitable for use as a specific SERS label.

## 3. Application of SERS Simultaneous Detection

In terms of food safety, SERS has been extensively utilized for detecting various contaminants. Thanks to the development of new SERS substrates with stronger Raman enhancement effects and the introduction of various Raman reporter molecules, SERS simultaneous detection has achieved more efficient analytical results. Whether it is labeled detection or label-free detection, it can detect pesticides, mycotoxins, antibiotics, food additives, and harmful microbes (Table 1). This ability is essential for ensuring food safety and quality, as it enables the identification and quantification of potentially harmful substances before they reach consumers. Currently, the common SERS simultaneous detection schemes include (1) label-free detection, (2) algorithm-assisted label-free detection, (3) labeled detection with antibody or aptamer functionalization (SERS signal encoding), (4) multi-detection at different positions assisted by lateral flow immunoassay (LFIA) (spatial isolation detection), and (5) SERS signal encoding detection at the same position assisted by LFIA.

**Table 1:** Applications of SERS technology for simultaneous detection of multiple food contaminants.

Contaminants	SERS Substrates	Strategy	Extra Technology	LOD	References
*P. aeruginosa*, *S. aureus*, *S. epidermidis*, *M. smegmatis*	AuNPs	Labeled	PCR; SERS reporter molecules: MBA, DTNB, MMC, TFMBA	100 copies of target gDNA	[78]
*E. coli*, *S. aureus*, *S. typhimurium*	Au@Ag@SiO_2_	Label-free	Machine learning	/	[79]
*E. coli*, *S. aureus*	AuNPs	Labeled	Aptamer; SERS reporter molecules: Cy3, ROX	10 CFU/mL	[80]
*E. coli*, *S. aureus*	Fe_3_O_4_@SiO_2_@Ag; Au-MPBA/DTNB@Ag	Labeled	Aptamer; SERS reporter molecules: DTNB, MPBA	1 CFU/mL	[81]
AFB1, OTA	Gold nanoparticles grafted onto silica photonic crystals	Labeled	Aptamer; LFIA, SERS reporter molecules: DTNB, NBA	0.36 pg/mL (AFB1), 0.034 pg/mL (OTA)	[82]
OTA, ZEN	Au@AgNPs	Labeled	Aptamer; SERS reporter molecules: MPY, MBN	0.94 pg/mL (OTA), 59 pg/mL (ZEN)	[83]
OTA, AFB1, DON	3D-Psi/AgNPs, AuNPs	Labeled	Antibody, SERS reporter molecules: NBA	3.35 pg/mL (OTA), 0.36 pg/mL (AFB1), 2.70 pg/mL (DON)	[84]
FB1, AFB1, ZEN	3D Au nanofilm	Labeled	Antibody, LFIA, SERS reporter molecules: DTNB	0.529 pg/mL (FB1), 0.745 pg/mL (AFB1), 5.90 pg/mL (ZEN)	[85]
IMI, PYR, AFB1	Au@AgNPs	Labeled	Antibody, LFIA, SERS reporter molecules: MBA	8.6 pg/mL (IMI), 97.4 pg/mL (PYR), 8.9 pg/mL (AFB1)	[86]
ACE, CBZ	Au@AgNPs	Labeled	Aptamer, SERS reporter molecules: PB, MBN	9.43 μg/kg (ACE), 9.17 μg/kg (CBZ)	[87]
CHL, IMI, OXY	Ag@AuNPs	Labeled	Antibody, LFIA, SERS reporter molecules: NTP	0.00015 ng/mL (CHL), 0.001 ng/mL (IMI), 0.0022 ng/mL (OXY)	[88]
CBZ, flumetralin	SiO_2_@Ag nanoparticles	Label-free	/	0.1 mg/kg (CBZ), 1 mg/kg (flumetralin)	[89]
ZIR, TBZ	Au@Ag^TGA^NRs	Label-free	/	0.003 mg/kg (ZIR), 0.028 mg/kg (TBZ)	[90]
TCP, OXA	Au@Ag@^2-MCE^NPs	Label-free	/	0.006 mg/kg (TCP in pear), 0.008 mg/kg (TCP in apple), 0.007 mg/kg (OXA in pear), 0.009 mg/kg (OXA in pear)	[91]
MG, CV	AgNPs	Label-free	/	100 fg/mL (MG), 1 pg/mL (CV)	[92]
ENR, ENX, CPX	Flexible cotton fabric/Ca-doped TiO_2_	Label-free	/	7.08 × 10^−9^ M	[93]
ENR, ENX	Flexible Cotton fabric/TiO_2_	Label-free	/	7.24 × 10^−7^ M	[94]
AM, TC, OFX	Ag-coated Au nanorod	Label-free	Chemometrics algorithms	1.8 × 10^−5^ μM (AM), 5 × 10^−6^ μM (TC), 1.5 × 10^−5^ μM (OFX)	[95]
ENR, ENX, CPX, CAP	(001) facet-supported TiO_2_ facet heterojunction	Label-free	/	6.6 × 10^−10^ M (ENR), 8.13 × 10^−10^ M (ENX), 6.17 × 10^−10^ M (CPX), 1.24 × 10^−9^ M (CAP)	[96]
ENR, ENX, CAP	TiO_2_/ZnO	Label-free	/	9.62 × 10^−10^ M (ENR), 8.57 × 10^−10^ M (ENX), 8.6 × 10^−10^ M (CAP)	[97]
TC, penicillin	Au@AgNPs	Labeled	Antibody, LFIA, SERS reporter molecules: DTNB, MBA	0.015 ng/mL and 0.010 ng/mL	[98]
CAP, TC	Au@AgNSs	Labeled	Antibody, SERS reporter molecules: DTNB, MBA	159.49 fg/mL (CAP), 294.12 fg/mL (TC)	[99]
NEO, LIN	AuNPs	Labeled	Antibody, LFIA, SERS reporter molecules: DTNB, ATP	0.33 pg/mL (NEO), 0.29 pg/mL (LIN)	[100]
ENR, MG, nitrofurazone, and Sudan I	Cu_2_O-Ag/AF-C_3_N_4_	Label-free	/	4.67 × 10^−4^ mg/L (ENR), 2.57 × 10^−5^ mg/L (MG), 5.7 × 10^−7^ mg/L (nitrofurazone), 6.92 × 10^−5^ mg/L (Sudan I)	[101]
Hg^2+^, Ag^+^	AuNPs	Labeled	cascade nucleic acid amplification, SERS reporter molecules: Cy3, ROX	4.4 aM (Hg^2+^), 9.97 aM (Ag^+^)	[102]
Sunset yellow, lemon yellow, carmine, erythrosine	Raspberry-like Ag nanoparticles	Label-free	Chemometrics algorithms	0.01 mg/L (colorant standard), 0.5 mg/L (black tea samples)	[103]
Ractopamine, salbutamol	AuNPs	Label-free	/	/	[104]

*P. aeruginosa*, *Pseudomonas aeruginosa*; *S. aureus*, *Staphylococcus aureus*; *S. epidermidis*, *Staphylococcus epidermidis*; *M. smegmatis*, *Mycobacterium smegmatis*; PCR, polymerase chain reaction; MBA, 4-mercaptobenzoic acid; DTNB, 5,5′-dithiobis-(2-nitrobenzoic acid); MMC, 7-mercapto-4-methylcoumarin; TFMBA, 2,3,5,6-tetrafluoro-4-mercaptobenzoic acid; E. coli, Escherichia coli; S. typhimurium, Salmonella typhimurium; Cy3, Cyanine 3; ROX, Carboxy-X-rhodamine; MPBA, 4-Mercaptophenylboronic acid; AFB1, aflatoxin B1; OTA, ochratoxin A; LFIA, lateral flow immunoassay; NBA, Nile blue A; ZEN, zearalenone; MPY, 4-mercaptopyridine; MBN, 4-(mercaptomethyl) benzonitrile; DON, deoxynivalenol; 3D-Psi, 3D porous silicon; FB1, fumonisin B1; IMI, imidacloprid; PYR, pyraclostrobin; ACE, acetamiprid; CBZ, carbendazim; PB, Prussian blue; CHL, chlorothalonil; OXY, oxyfluorfen; NTP, 4-nitrothiophenol; ZIR, ziram; TBZ, thiabendazole; TGA, thioglycolic acid; TCP, thiacloprid; OXA, oxamyl; 2-MCE, 2-mercaptoethylamine; MG, malachite green; CV, crystal violet; ENR, enrofloxacin; ENX, enoxacin; CPX, ciprofloxacin; CAP, chloramphenicol; AM, amoxicillin; TC, tetracycline; OFX, ofloxacin; NEO, neomycin; LIN, lincomycin; ATP, 4-aminothiophenol; AF-C_3_N_4_, oxygen-linked graphitic carbon nitride.

### 3.1. Detection of Harmful Microbes

Harmful microbes, especially pathogenic bacteria, are responsible for foodborne illnesses and can be transmitted through contaminated food. A wide variety of foodborne pathogenic bacteria are capable of surviving and proliferating in diverse environments, thereby posing a substantial risk to human health [105].

**Figure 4:**
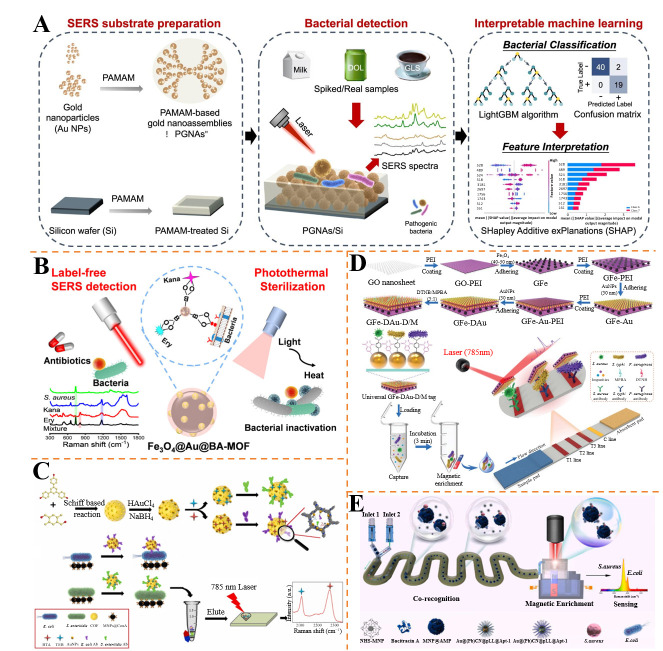
(**A**) SERS substrate preparation and simultaneous detection scheme diagram of four pathogenic bacteria [106]. (**B**) Schematic diagrams of capture of multiple contaminants, label-free SERS detection, and photothermal sterilization [107]. (**C**) Schematic diagram of the preparation process of a novel COF Raman tag and its application in simultaneous immune SERS detection of *E. coli* and *Salmonella enteritidis* [108]. (**D**) Schematic of the preparation of film-type GFe–DAu–D/M probes and GFe–DAu–D/M–ICA for the multiplex detection of *S. aureus*, *S. typhi*, and *P. aeruginosa* [109]. (**E**) Schematic diagrams of co-recognition, enrichment, and sensing all-in-one strategy coupling with integrated flexible microfluidics and magnetic control slider SERS platform [112].

Qiu et al. developed a dendrimer-based SERS platform integrated with class-incremental learning (CIL) using LightGBM algorithm for rapid detection of four pathogens (*E. coli*, *Salmonella Paratyphi B*, *P. aeruginosa*, *S. aureus*) [106] (Figure 4A). The PAMAM-based gold nano-assemblies on silicon wafer (PGNAs/Si) exhibited high SERS activity with LOD of 10 CFU/mL in water, food, and dietary supplement matrices. The CIL model achieved classification accuracy over 93.44%. This approach combines efficient bacterial capture with machine learning for robust, interpretable multiplex detection in complex matrices.

Shang et al. constructed a multifunctional SERS substrate (Fe_3_O_4_@Au@BA-MOF) for dual detection of pathogens and antibiotics, along with photothermal sterilization [107] (Figure 4B). The boronic acid-modified composite enabled specific capture of bacteria via cis-diol recognition, with an LOD of 10 CFU/mL for *E. coli* and *S. aureus*. Under near-infrared light, the substrate achieved 100% and 99.3% bactericidal rates within 8 min, respectively. Real samples (milk, chicken) showed recoveries of 96~110%, highlighting its potential for integrated detection and decontamination in food safety and clinical settings.

Yang et al. developed a SERS immunosensor using covalent organic frameworks (COF) as biologic interference-free Raman tag containers for multiplex detection of foodborne pathogens [108] (Figure 4C). Lectin-functionalized magnetic nanoparticles (MNPs@ConA) captured pathogens, forming MNPs@ConA/pathogen/COF@Raman tag complexes. Elution of reporters enabled detection of characteristic signals at 2271 cm^−1^ and 2113 cm^−1^, with limits of detection of 10^1^ CFU/mL for each strain. The strategy avoided background interference from biological matrices, achieving recoveries of 86.32~112.47% in food samples, highlighting its reliability for simultaneous pathogen analysis.

Li et al. introduced a 2D film-like magnetic SERS tag (GFe-DAu-D/M) for multiplex detection of *S. aureus*, *P. aeruginosa*, and *S. typhimurium* [109] (Figure 4D). The tag integrated GO nanosheets, Fe_3_O_4_ NPs, and dual AuNP layers with MPBA/DTNB reporters, enabling magnetic enrichment and SERS signal amplification. With an LOD of 10 CFU/mL and linear ranges up to 10^5^ CFU/mL, the method showed 98% capture efficiency and excellent specificity in urine samples.

Yu et al. developed dual-mode aptasensors using AuNPs and silver-core gold-shell (Ag@Au) nanoparticles for simultaneous detection of three foodborne pathogens: *E. coli*, *S. aureus*, and *Listeria monocytogenes* [110]. The AuNP-based lateral flow aptasensor enabled visual detection with an LOD of 20~30 CFU/mL, while the Ag@Au NP-based SERS aptasensor achieved ultra-sensitive quantification with an LOD as low as 2 CFU/mL. The SERS platform leveraged the plasmonic enhancement of Ag@Au heterostructures and aptamer specificity, providing linear ranges of 10^2^~10^7^ CFU/mL for all three pathogens. In real samples (milk and chicken), recoveries were in the range of 89.87~117.35%, with relative standard deviation (RSD) < 8.6%. This dual-modality strategy combines the simplicity of visual readout with the high sensitivity of SERS, offering a versatile solution for rapid screening and precise quantification in food safety monitoring.

Bai et al. presented a SERS-based sandwich immunoassay platform for interference-free simultaneous detection of *E. coli* and *S. aureus* using magnetic beads and dual-coded Raman silent probes [111]. The system employed aptamer-functionalized AuNPs labeled with triple-bond reporters (C≡C and C≡N) at 2105 cm^−1^ and 2227 cm^−1^, avoiding spectral overlap with complex matrices. Magnetic beads enabled efficient enrichment, reducing background noise and improving sensitivity, with LODs of 10 CFU/mL for *E. coli* and 25 CFU/mL for *S. aureus*. In bottled water and milk samples, recoveries were in the range of 93.8~110%, with RSDs < 6%. The platform’s specificity was validated against interfering pathogens (e.g., *Salmonella, Listeria*), demonstrating its robustness for multiplex detection in real-world food matrices with minimal cross-reactivity.

Huo et al. introduced a co-recognition, enrichment, and sensing (CES) platform integrating flexible microfluidics, magnetic enrichment, and silent SERS for simultaneous analysis of *S. aureus* and *E. coli* [112] (Figure 4E). Antimicrobial peptide (AMP)-functionalized magnetic nanoparticles (MNPs) captured bacteria, while aptamer@Au@PB NPs served as SERS tags with distinct silent Raman peaks (2139 cm^−1^ and 2197 cm^−1^). The modular microfluidic device with a magnetic control slider enabled rapid switching between enrichment and detection, achieving LODs of 14 CFU/mL and 18 CFU/mL, respectively, with linear ranges of 50~1600 CFU/mL. In spiked milk and chicken samples, recoveries were in the range of 85.6~112.3% (RSD < 8.6%), and results correlated well with ELISA (relative errors < 4.3%). This all-in-one strategy offers high specificity and anti-interference capability, suitable for on-site food safety and clinical diagnostics.

### 3.2. Detection of Mycotoxins

Mycotoxins are toxic secondary metabolites synthesized by various fungal species and are frequently present in foodstuffs such as grains, fruits, and vegetables [113,114]. These toxins can form under specific temperature and humidity conditions, which are commonly encountered during food processing, storage, and transportation. Recent advancements in nanotechnology have facilitated the development of various SERS substrates, enabling the simultaneous coupling of multiple Raman reporting molecules for SERS signal encoding and making this technology more applicable for multiplex detection in actual samples [115]. Therefore, SERS has become an active research field for detecting mycotoxins, including ZEN, aflatoxin, DON, and T-2 toxin [116,117].

**Figure 5:**
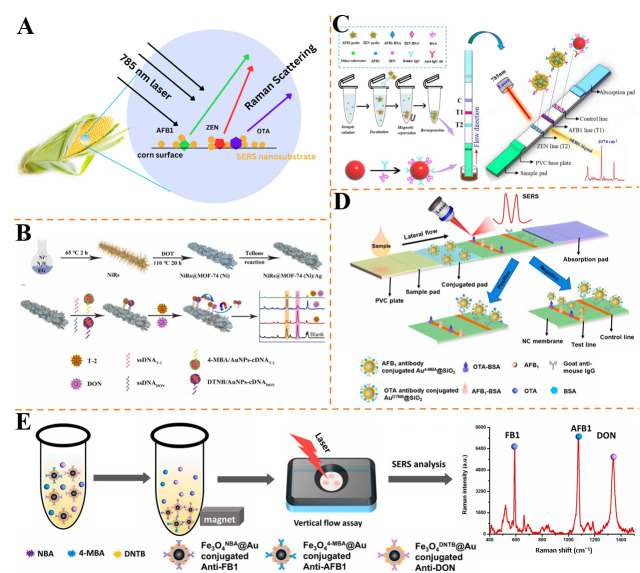
(**A**) Schematic illustration of the developed method used to detect mycotoxin analytes on corn [121]. (**B**) Schematic illustration of preparation of NiRs@MOF-74 (Ni)/Ag for simultaneous SERS analysis of T-2 and DON [125]. (**C**) The design of Fe_3_O_4_@PEI/Au^MBA^@Ag-MBA-based SERS-LFIA strips for simultaneous detection of two mycotoxins [126]. (**D**) LFIA strip assembly and the principle of competitive SERS-LFIA for simultaneous detection of AFB1 and OTA [127]. (**E**) Schematic of FB1, AFB1, and DON detection with the VFA biosensor [129].

Aflatoxins are a group of mycotoxins, comprising at least 18 distinct types, with AFB1 being the most toxic and carcinogenic. As such, AFB1 represents a significant regulatory concern in agricultural products [118]. ZEN, identified as an endocrine disruptor, has been shown to impair organ development, cause reproductive disorders, and lead to digestive dysfunction in animals, thereby posing substantial healthy risks [119]. Humans are similarly at risk of ZEN exposure through the consumption of contaminated food, underscoring the importance of effective detection to protect public health [120]. As illustrated in Figure 5A, Gabbitas et al. developed a label-free SERS method for the simultaneous detection of three mycotoxins—AFB1, ZEN, and OTA [121]. Using AuNPs as the SERS substrate, the method leveraged the intrinsic Raman fingerprints of each mycotoxin for discrimination, achieving LODs of 10 μg/kg (32 nM) for AFB1, 20 μg/kg (64 nM) for ZEN, and 100 μg/kg (248 nM) for OTA. Multivariate statistical analysis (partial least squares regression) enabled quantitative prediction of mycotoxin concentrations up to 1.5 mg/kg, with correlation coefficients of 0.74~0.89. The entire sampling process took less than 30 min, demonstrating its potential for rapid, multiplex screening in food safety applications without complex labeling or sample pretreatment.

T-2 toxin, the most potential and toxic type A trichothecene, is produced by various fungi and is commonly found in corn, oats, barley, wheat, and animal feed under cold or humid storage conditions. Its impact is not only detrimental to livestock but also poses a risk to human health [122]. Huang et al. fabricated a gold-grafted melamine sponge (AuSp) as a stable SERS substrate for enzyme-linked immunoassay of ZEN and T-2 toxin [123]. The AuSp integrated Au nanoparticles for Raman enhancement and melamine sponge for structural stability, achieving LODs of 1 μg/kg for ZEN and 0.05 μg/kg for T-2 toxin, with linear ranges of 5~100 and 0.1~20 μg/kg, respectively. The method showed recoveries of 85~120% in cereal samples, with relative standard deviations below 7.0%. After one-year storage, the signal strength decreased by less than 10%, highlighting its long-term stability. This approach simplifies substrate preparation and reduces sample consumption, suitable for on-site mycotoxin detection.

DON contamination not only compromises grain quality but also induces a range of harmful effects, including vomiting, anorexia, immunotoxicity, and disruption of growth and reproduction [124]. Ge et al. developed a magnetic SERS aptasensor using NiRs@MOF-74(Ni)/Ag composites for simultaneous detection of T-2 and DON [125] (Figure 5B). The substrate integrated magnetic nickel rods, MOF-74(Ni) for adsorption, and Ag nanoparticles for plasmonic enhancement, achieving LODs of 0.15 μg/L for T-2 and 0.08 μg/L for DON, with linear ranges of 0.5~750 and 0.3~750 μg/L, respectively. In spiked corn samples, recoveries ranged approximately from 80.4 to 116%, with relative errors <7.7% compared to HPLC-MS. The method’s magnetic separation and high selectivity against interferents (e.g., AFB1, ZEN) make it suitable for complex food matrices.

OTA is a common mycotoxin found in grains such as corn and wheat, alongside AFB1 and ZEN. These mycotoxins not only degrade the quality of the grains but also pose a potential health threat. LFIA offers advantages such as portability, rapid results, low cost, and ease of use, making it especially suitable for on-site testing. When integrated with SERS technology, LFIA can meet the demands for rapid detection of trace mycotoxins in complex matrices. For example, Yin et al. presented a dual-functional magnetic SERS-LFIA using core–interlayer–satellite (Fe_3_O_4_@PEI/Au^MBA^@Ag) nanocomposites as SERS tags [126] (Figure 5C). The tags integrated magnetic enrichment and plasmonic enhancement, enabling simultaneous detection of AFB1 and ZEN in corn with LODs of 0.095 μg/kg and 1.896 μg/kg, respectively, and linear ranges of 0.1~10 μg/kg (AFB1) and 4~400 μg/kg (ZEN). In spiked samples, recoveries ranged from 87.0% to 112.0% (RSD < 10%), and results correlated well with HPLC. The method achieved visual and SERS dual-readout within 20 min, overcoming matrix interference and demonstrating suitability for on-site screening. Chen et al. presented a SERS-LFIA using Au@SiO_2_ nanotags encoded with MBA) and DTNB for simultaneous detection of AFB1 and OTA [127] (Figure 5D). The dual-labeled nanotags enabled competitive immunoassay on a single test line, achieving ultra-low LODs of 0.24 pg/mL for AFB1 and 0.37 pg/mL for OTA, far below regulatory limits. In spiked corn, rice, and wheat samples, recoveries ranged approximately from 87.0 to 112.0% for OTA and 91.0 to 104.8% for AFB1, with high reproducibility (RSD < 6%). The method combines the simplicity of LFIA with SERS sensitivity, offering a portable solution for multiplex mycotoxin monitoring in food matrices.

Fumonisin is a secondary metabolite produced by the fungi *Fusarium verticillioides*, *Fusarium proliferatum*, and other related species, and it is usually present as a pollutant in corn and corn by-products. Fumonistin B, including FB1, FB2, and FB3, is the most abundant natural fumonistin, among which FB1 is the main and most toxic form [128]. Chen et al. developed a vertical flow immunoassay (VFA) using Fe_3_O_4_@Au magnetic SERS nanotags for simultaneous detection of FB1, AFB1, and DON [129] (Figure 5E). The core-shell tags, functionalized with NBA, 4-MBA, and DNTB reporters, enabled magnetic enrichment and specific recognition, achieving LODs of 0.053, 0.028, and 0.079 pg/mL, respectively. In spiked wheat samples, recoveries ranged approximately from 86.6 to 108.4% (RSD < 13.48%), matching ELISA results. The method’s rapid workflow (20 min) and high sensitivity make it suitable for early-stage screening of mycotoxins in agricultural products.

### 3.3. Detection of Pesticides

While the widespread use of pesticides has significantly boosted agricultural productivity [130], it has also led to substantial pesticide residues, which pose considerable risks to both ecosystems and human health [131]. As a result, there is an urgent need for the development of simple, rapid, sensitive, and reliable methods for monitoring pesticide residues in food products. SERS has emerged as a powerful technique for pesticide residue detection, owing to its non-destructive nature, high sensitivity, and rapid analysis capabilities [132,133].

**Figure 6:**
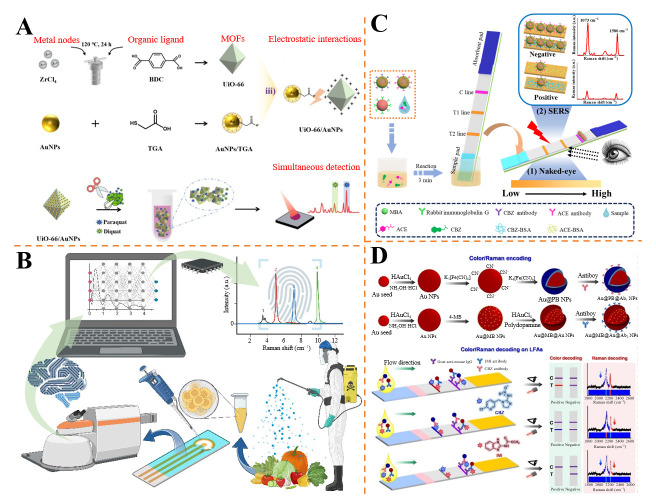
(**A**) Illustration of the synthesis and detection process of UiO-66/AuNPs SERS substrate [134]. (**B**) Schematic diagram of the combination of SERS and machine learning for the detection of multiple pesticide residues in agricultural products [138]. (**C**) Illustration of SERS micro-well lateral flow dual-modal immunosensor detection process [139]. (**D**) Schematic illustration of the synthesis process of the color- and Raman-encoded nanoprobes and the competitive LFIA based on the designed nanoprobes for simultaneous detection of CBZ and IMI through either the color decoding or Raman decoding methods [140].

Pu et al. fabricated UiO-66/AuNPs SERS substrates via thioglycolic acid-mediated electrostatic assembly for paraquat (PRQ) and diquat (DQT) detection in cabbage [134] (Figure 6A). PRQ exhibited characteristic peaks at 1649 cm^−1^ (C=N stretching) and 1190 cm^−1^ (C=C bending), while DQT showed signals at 1578 cm^−1^ (aromatic ring/C=N coupling) and 1072 cm^−1^ (aromatic ring/C–H bending). The method achieved LODs of 3 μg/L (PRQ) and 6 μg/L (DQT), with recoveries of 92.65~118.34% in cabbage. Notably, it distinguished mixtures with 100-fold concentration differences, highlighting its utility for trace analysis in leafy vegetables. Ma et al. synthesized Au@Ag core–shell nanoparticles as SERS substrates for simultaneous detection of TRM and ACE in apple/orange juices [135]. ACE exhibited distinct peaks at 635 cm^−1^ (C–C–C wagging), 827 cm^−1^ (N–C=N ring breathing), and 1112 cm^−1^ (N–C stretching), while TRM showed signals at 1385 cm^−1^ (C–N stretching), 565 cm^−1^ (S–S stretching), and 926 cm^−1^ (N–CH_3_/C=S stretching). The method showed linear ranges of 5~100 μM (ACE) and 0.5~10 μM (TRM), with LODs of 1.22 μM and 0.076 μM, respectively. Recoveries in juice samples were in the range of 90.2~122.12%, demonstrating its feasibility for on-site monitoring of mixed pesticides in complex food matrices.

In addition, spectral data often contains noise originating from the sample substrate, which can complicate analysis. Chemometric algorithms play a crucial role in extracting meaningful information from these spectra. For instance, Wang et al. developed sulfhydryl-functionalized Fe_3_O_4_@SiO_2_@Ag-SH magnetic SERS substrates for qualitative and quantitative analysis of four benzimidazoles (CBZ, benomyl, thiophanate-methyl, TBZ) in corn [136]. Characteristic Raman peaks of benzimidazoles were identified at 619 cm^−1^, 725 cm^−1^, 1008 cm^−1^, 1272 cm^−1^, 1592 cm^−1^, and 1478 cm^−1^. The partial least squares discriminant analysis (PLS-DA) model achieved a recall rate >99.17%, while support vector machine regression (SVR) yielded LODs of 0.055~0.093 mg/L and recoveries of 85.6~107.5%. The method showed no significant difference from HPLC (*p* > 0.05), validating its utility for rapid pesticide residue screening in food matrices. Recent advancements in deep learning have significantly enhanced the field of chemometrics, enabling more effective self-learning and modeling of spectroscopic data [137]. For example, Hegde et al. introduced SERS Former-2.0, a transformer architecture combined with Au@Ag core–shell nanoparticles, for simultaneous identification and quantification of pesticide mixtures in fresh produce [138] (Figure 6B). Key Raman signatures included thiabendazole at 1649 cm^−1^ (C=N stretching) and 1536 cm^−1^ (–CH_2_ bending/C–N coupling) and carbophenothion at 1234 cm^−1^ (C=C bending) and 1271 cm^−1^ (C–C structural deformation). The model achieved exceptional multilabel classification performance (accuracy = 0.999, F1 score = 0.992) and multiregression accuracy (*R*^2^ = 0.804), resolving spectral dominance issues in mixed samples and enabling robust detection in real-world scenarios.

Ma et al. presented a dual-modal SERS immunoassay strip for rapid detection of ACE and CBZ in fruits [139] (Figure 6C). The strip employed double-layer MBA-labeled Au^MBA^@Ag^MBA^ NPs conjugated with target antibodies as signal probes and Au NPs adsorbed with rabbit IgG as indicating probes. The dual-modal strategy allowed semi-quantitative naked-eye detection via colorimetric comparison and quantitative analysis via SERS. The linear ranges were 0.3~2 μg/kg for ACE and 3~30 μg/kg for CBZ, with LODs of 0.27 μg/kg and 1.71 μg/kg, respectively. Spiked recovery experiments in apple and orange samples showed 93.86~105.64% (ACE) and 92.62~102.30% (CBZ) recoveries, consistent with HPLC results. This portable strip enables on-site monitoring of mixed pesticide residues with high sensitivity and reliability. Wang et al. reported a dual-encoded LFIA for simultaneous detection of CBZ and imidacloprid (IMI) [140] (Figure 6D). The system utilized Au@PB NPs (blue-colored, Raman peak at 2151 cm^−1^) and Au@MB@Au NPs (red-colored, Raman peak at 2223 cm^−1^) as immunoprobes, encoding both colorimetric and Raman signals in the Raman-silent region (1800~2800 cm^−1^) to avoid background interference. In the competitive LFIA, the T-line color shifted from purple to red/blue or colorless depending on pesticide presence, enabling naked-eye semi-quantification (LODs: CBZ 1.20 ng/mL, IMI 1.32 ng/mL). Raman detection achieved lower LODs (CBZ 0.03 ng/mL, IMI 0.04 ng/mL), with recoveries in cucumber, apple, and lake water samples ranging from about 74.6 to about 117.5% and with good agreement with HPLC (*R*^2^ > 0.99). This method combines visual and spectral analysis for robust multiplex detection.

Lu et al. developed a DNA backbone-structured Ag@Au nano-tetrahedron biosensor for simultaneous detection of profenofos, ACE, and CBZ using SERS [141]. The nano-tetrahedron was constructed by embedding aptamers specific to the three pesticides into the DNA framework, with Ag@AuNPs modified by distinct Raman signaling molecules (ATP, NTP, methoxybenzyl mercaptan) at the tetrahedral corners. Upon pesticide recognition, the DNA backbone deformation brought Ag@Au NPs closer, forming SERS hotspots and enhancing Raman signals. The biosensor exhibited ultra-low LODs of profenofos (0.0021 ng/mL), ACE (0.0046 ng/mL), and CBZ (0.0061 ng/mL) and high sensitivity. Recovery tests in food and environmental samples showed rates of 79.5~98.7%, and the results correlated well with HPLC-MS (*R*^2^ > 0.99), demonstrating its practical applicability for multiplex pesticide analysis.

### 3.4. Detection of Antibiotic

Antibiotics are widely used in aquaculture, livestock, poultry production, and agriculture to boost economic output [142]. As a result, residues of these drugs are commonly found in a variety of food products, including meat, fish, milk, eggs, and fruits. These residues can accumulate in the human body through the food chain, potentially leading to organ damage and contributing to health issues such as anemia and cardiovascular diseases [143]. Therefore, it is critical to develop reliable methods for monitoring and analyzing veterinary drug residues in aquatic products to safeguard food safety.

**Figure 7:**
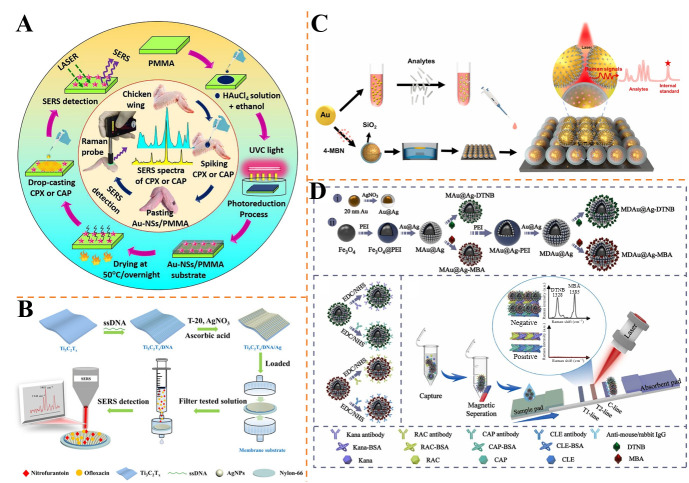
(**A**) Schematic of the fabrication process of Au-NSs/PMMA SERS substrate and SERS on-site sensing on chicken wings [144]. (**B**) Schematic diagram of the preparation of SERS film substrates and the quantitative trace of multiple antibiotic residues [145]. (**C**) Illustration of the SERS calibration substrate with a silent region internal standard for simple and reliable quantitative detection [146]. (**D**) Schematic representation of synthesis of (**i**) 24 nm Au@Ag NPs and (**ii**) multilayered MDAu@Ag tags with dual layers of Au@Ag and Raman dyes, preparation of immuno-MDAu@Ag SERS tags, and design of MDAu@Ag-based SERS-LFA for simultaneous detection of four veterinary drugs [150].

Barveen et al. developed a flexible SERS substrate by photochemically synthesizing gold nanostars (AuNSs) on poly(methyl methacrylate) (PMMA) films using ethanol as a reducing agent [144] (Figure 7A). The AuNSs/PMMA substrate integrated plasmonic hotspots from sharp nanospikes with mechanical flexibility, enabling in situ detection of CPX and CAP on curved surfaces like chicken wings. With an enhancement factor of 2.03 × 10^9^, the substrate achieved ultra-low LODs of 3.41 × 10^−11^ M for CAP and 7.77 × 10^−10^ M for CPX. The method showed high reproducibility (RSD < 7.32%) and multiplexing capability, with recoveries in spiked samples approximately ranging from 85.6 to 112.3%. Its transparency and recyclability via UV-induced photodegradation make it suitable for on-site food safety monitoring.

Yu et al. developed a Ti_3_C_2_T_x_/DNA/Ag membrane substrate for SERS, enabling simultaneous quantification of trace nitrofurantoin (NFT) and OFX in aquatic samples [145] (Figure 7B). The substrate integrated electromagnetic and chemical enhancement effects, achieving multitarget separation, enrichment, and in situ detection. It showed good uniformity, reproducibility, and stability, with SERS enhancement factors of 1 × 10^5^ for NFT and 3 × 10^5^ for OFX. The linear ranges were 20~500 μg/L for NFT and 60~800 μg/L for OFX, with LODs of 12.0 and 35.0 μg/L, respectively. Recoveries in aquatic samples were in the range of 88~107%, and relative errors compared to HPLC were from −9.8 to 5.3%, verifying its accuracy for rapid, high-throughput analysis of antibiotic residues in complex matrices.

Chen et al. prepared a self-calibrating SERS substrate using Au@4-MBN@SiO_2_ nanoparticles assembled on silicon wafers, enabling simultaneous detection of penicillin potassium (PP), tetracycline hydrochloride (TCH), and levofloxacin (LEV) [146] (Figure 7C). The chemically inert SiO_2_ shell ensured long-term stability (over 24 weeks) and minimized matrix interference, while the internal standard (IS) band at 2223 cm^−1^ from 4-MBN corrected signal fluctuations. The method achieved LODs of 26.9 nM (PP), 28.2 nM (TCH), and 2.4 nM (LEV), with linear ranges up to 100 mg/L and recoveries of 87~112% in lake water. Principal component analysis (PCA) confirmed 100% sensitivity and specificity for multicomponent analysis, demonstrating its reliability for environmental antibiotic screening.

Yuan et al. proposed a deep learning-driven SERS method for simultaneous quantification of CPX, doxycycline, and LEV in municipal lake water [147]. By integrating convolutional neural networks (CNNs) with a non-negative elastic network (NN-EN), the approach analyzed complex Raman spectra to predict antibiotic ratios in mixed solutions. The LODs reached 10^−7^ M, with linear dynamic ranges spanning five orders of magnitude. Real lake water samples showed recoveries of 88.8~111.3% and RSDs below 16%, demonstrating its capability for rapid, multiplex environmental monitoring with high accuracy and resistance to matrix interference.

Shi et al. established a thin-layer chromatography (TLC)-SERS method for simultaneous detection of 14 nitroimidazoles in pork samples [148]. Using gold nanoparticles (AuNPs) as SERS substrates, the method combined theoretical Raman spectra calculations with experimental validation, achieving an LOD of 0.1 mg/L. Twelve nitroimidazoles were successfully separated by optimized TLC conditions, while chemometrics (PCA) resolved unseparated metronidazole and ronidazole. Spiked pork samples showed recoveries of 80~110% and RSDs of 0.74~16.70%, highlighting its utility for rapid, multiplex screening of nitroimidazole residues in complex food matrices.

Tang et al. proposed a SERS-activated molecularly imprinted capillary sensor (SERS-CP-MI) for simultaneously detecting ampicillin (APC) and CAP in chicken and milk [149]. The sensor integrated AgNPs-modified capillaries with dual-template imprinted polymers (TMIP), achieving LODs of 1.3 × 10^−7^ M for APC and 1.8 × 10^−7^ M for CAP. The capillary’s siphon effect enabled microvolume analysis (10 μL), with recoveries in spiked samples in the range of 97.1~103.8% and RSDs < 10%. This method combines high selectivity of molecular imprinting with SERS sensitivity for efficient multi-residue detection in food.

Tu et al. introduced multilayered magnetic-core dual-shell SERS tags (MDAu@Ag) into LFIA for simultaneous detection of four veterinary drugs (kanamycin, ractopamine (RAC), clenbuterol (CLE), CAP) [150] (Figure 7D). The MDAu@Ag, with precise nanogaps and magnetic enrichment, enabled dual signal amplification, achieving LODs as low as 0.52 pg/mL (kanamycin) and 6.2 pg/mL (CLE). The dual-test-line design allowed multiplex detection within 35 min, with recoveries in pork and lake water samples exceeding 85% and RSDs < 8%. This method provides a stable, ultrasensitive point-of-care testing tool for complex environmental and food matrices.

Wu et al. developed a magnetic Fe_3_O_4_@Au nanotag-based colorimetric/SERS dual-readout LFIA for CLE and RAC [151]. The dual-functional tags integrated magnetic enrichment and SERS signal amplification, achieving LODs of 7.8 pg/mL (CLE) and 3.5 pg/mL (RAC) via SERS and 1 ng/mL (CLE) and 0.33 ng/mL (RAC) via colorimetry. Recoveries in pork, beef, and mutton ranged approximately from 89.63 to 110.6% with RSDs < 10%, demonstrating high accuracy and anti-interference capability for on-site food safety testing.

### 3.5. Detection of Other Contaminants

Other food contaminants such as food additives, colorants, and bacterial toxins pose significant risks due to their toxicity, carcinogenicity, or mutagenicity, potentially causing both acute and chronic health effects. SERS technology offers a promising solution for the simultaneous detection of these substances, leveraging its ability to obtain fingerprint spectra with high sensitivity and accuracy.

Ge et al. developed a rapid and sensitive SERS method for simultaneous detection of three polychlorinated phenols in water samples using aggregated silver nanoparticles (AgNPs) induced by Na_2_SO_4_ as the substrate [152]. The electromagnetic field enhancement was verified by finite difference time domain (FDTD) simulations, and PCA was employed to resolve overlapping Raman peaks of structural analogs. Under optimized conditions, the LODs were 0.27 mg/L for 2,4-dichlorophenol, 0.09 mg/L for 2,4,5-trichlorophenol, and 0.10 mg/L for 2,3,4,6-tetrachlorophenol. The method showed recoveries of 80.4~114.0% with RSDs of 0.4~10.7%, demonstrating its high-throughput capability and accuracy for screening similar-structured contaminants in water and pesticide samples.

**Figure 8:**
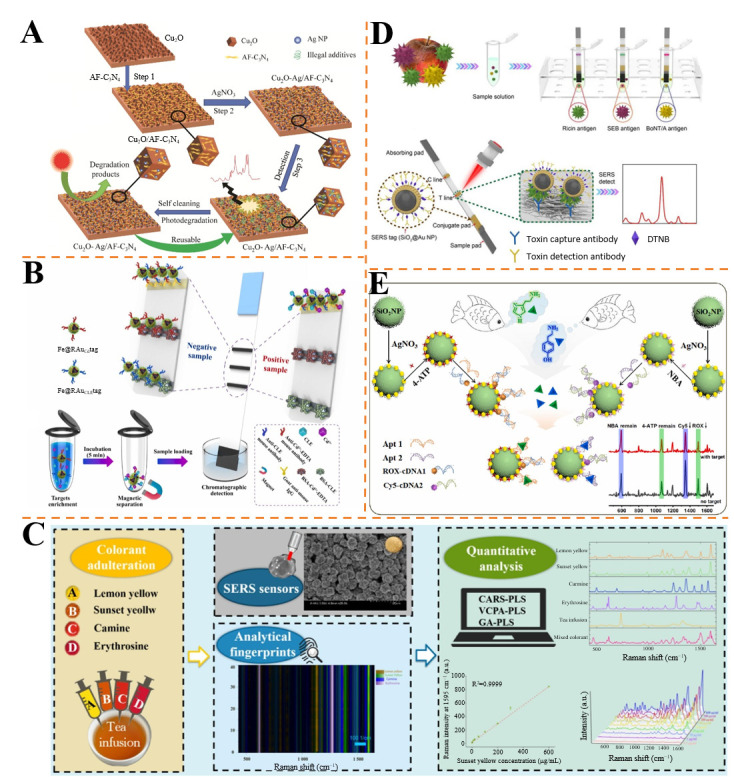
(**A**) Schematic illustration of the substrate fabrication and SERS detection for illegal additives [101]. (**B**) Schematic of the proposed SERS-LFIA technique for the simultaneous and ultrasensitive monitoring of CLE and Cd^2+^ in complex samples [156]. (**C**) Schematic diagram of hemp spherical silver nanoparticles used as SERS substrates for simultaneous detection of four colorants [159]. (**D**) Schematic illustration and detection procedure of the simultaneous detection of Ricin, SEB, and BoNT/A by using the SERS–LFIA strips [160]. (**E**) Schematic illustration of ratiometric SERS aptasensor for simultaneous detection of HIS and TYR based on the SiO_2_@Ag NPs active substrate [161].

As shown in Figure 8A, Liu et al. developed a reusable SERS substrate of Cu_2_O-Ag/AF-C_3_N_4_ by modifying Cu_2_O with AF-C_3_N_4_ nanosheets followed by reaction with AgNO_3_ solution [101]. This substrate integrated SERS enhancement from Ag nanoparticles and photocatalytic self-cleaning from AF-C_3_N_4_, enabling rapid and simultaneous detection of multiple illegal additives (ENR, MG, nitrofurazone, and Sudan I) in feed and food samples. The LODs for these additives were 4.67 × 10^−4^ mg/L, 2.57 × 10^−5^ mg/L, 5.7 × 10^−7^ mg/L, and 6.92 × 10^−5^ mg/L, respectively. It exhibited good uniformity and reproducibility, with RSDs of 6.74% and 4.85%, and retained over 80% of the initial Raman signal after four reuse cycles. Recoveries in actual samples ranged from 86.00% to 108.54%, demonstrating its potential for efficient and cost-effective food and feed safety monitoring.

Moreover, heavy metal ions, which accumulate in organisms through food webs within ecological systems, present substantial risks to animals, plants, and humans [153]. Public concern over the contamination of food with heavy metals has grown significantly in recent decades [154]. Upon absorption, these ions can bind to proteins, leading to their inactivation and impairing the health and safety of organisms. Consequently, rapid, cost-effective, and precise detection methods for heavy metal ions are crucial for both human health monitoring and environmental protection [155]. Dong et al. developed a Raman reporter-embedded magnetic SERS tag (Fe@RAu) for simultaneous immunochromatographic detection of Cd^2+^ and clenbuterol (CLE) in complex samples [156] (Figure 8B). The tag integrated DTNB as a Raman probe and magnetic beads for rapid enrichment, achieving LODs of 1.88 pg/mL for Cd^2+^ and 0.48 pg/mL for CLE—2000-fold more sensitive than traditional AuNP-based ICAs. With recoveries of 84.36~122.14% and RSDs < 11.2%, the method enabled direct analysis of spiked milk and pork extracts, showcasing its potential for on-site food safety screening. Jin et al. proposed a data fusion strategy combining SERS and fluorescence spectra to simultaneously quantify potassium sorbate and Pb^2+^ in *Tricholoma matsutakes* [157]. Using CNNs for decision-level fusion, the model achieved *R*^2^ values of 0.9963 and 0.9934, with RMSEs of 0.0712 g/kg and 0.0795 mg/kg, respectively. The method overcame low-concentration detection limitations by leveraging spectral complementarity, with LODs of 2.35 mg/kg and 9.72 μg/kg, demonstrating high accuracy in real matsutake samples.

Sun et al. presented a simple and low-cost SERS substrate using Au nanorod-incorporated melamine foam (AuNR-MF) for simultaneous detection of rhodamine B and basic orange II in chilli products [158]. Density functional theory (DFT) calculations analyzed the molecular vibration and enhancement mechanisms, confirming the electromagnetic contribution from AuNR aggregates. The LODs were 0.1 μg/mL for rhodamine B and 0.5 μg/mL for basic orange II, with recoveries of 82.3~108.5% in spiked samples. The substrate’s uniformity (RSD = 6.43%) and rapid analysis (30 s per sample) outperformed traditional HPLC methods for multiplex dye detection.

Li et al. synthesized hemp spherical AgNPs as SERS substrates for rapid detection of four colorants in black tea [159] (Figure 8C). The AgNPs showed an enhancement factor of 10^8^ and stability over 60 days. Using competitive adaptive weighted sampling–partial least squares (CARS-PLS), the method achieved LODs of 0.1~1 ng/mL, with prediction values (*R*^2^) of 0.88~0.99 and recoveries of 91.87~106.5%. The sensor differentiated mixed colorants via characteristic Raman peaks, offering a cost-effective approach for adulteration screening without sample pretreatment.

Jia et al. fabricated SiO_2_@Au nanoparticle-based SERS-LFIA strips for simultaneous detection of ricin, staphylococcal enterotoxin B (SEB), and botulinum neurotoxin type A (BoNT/A) [160] (Figure 8D). The strips achieved LODs of 0.1 ng/mL (ricin/BoNT/A) and 0.05 ng/mL (SEB), 100-fold more sensitive than traditional colloidal gold LFIA. With good specificity (no cross-reactivity with cholera/SEA) and repeatability (RSDs < 5%), the method enabled rapid (15 min) multi-toxin screening in food and environmental samples, addressing bioterrorism and contamination risks.

Duan et al. presented a ratiometric SERS aptasensor using SiO_2_@Ag core/shell nanoparticles for simultaneous detection of histamine (HIS) and tyramine (TYR) [161] (Figure 8E). By embedding ATP and NBA as internal standards, the sensor achieved LODs of 0.2 ng/mL HIS and 0.05 ng/mL TYR via I_1503_/I_1079_ and I_1358_/I_592_ ratios. With recoveries of 95.3~106.2% in spiked fish samples and RSDs < 4.2%, the method overcame matrix interference, offering a robust platform for biogenic amine analysis in animal-derived foods.

## 4. Challenges and Outlook

With the vigorous development of SERS technology in the detection of harmful contaminants in food, significant progress has been made from the detection of single contaminants to that of multiple contaminants. The sensing method based on SERS technology has the advantages of rapid detection, high sensitivity, non-destruction, less sample preparation, resistance to water interference, and reduced photobleaching. However, the current challenges still need to be solved in the simultaneous detection of multiple substances:

(1) The stability and reproducibility of SERS substrate materials need to be improved. The liquid substrate (such as gold/silver nano-sols) has poor signal reproducibility due to the disorder of local aggregation of nanoparticles. Although the stability of solid-state substrates is enhanced through regular arrangement, the preparation process is complex and it is difficult to standardize production. Developing new composite nanomaterials and improving the base preparation process can enhance the uniformity and stability of the nanoparticle hotspots. One potential method involves combining graphene or its derivatives with noble metal nanoparticles, such as preparing reduced graphene oxide/silver nanoparticles (RGO/AgNPs) composite substrates. This composite structure generates a chemical enhancement mechanism through charge transfer, while the surface plasmon resonance of the silver nanoparticles provides electromagnetic enhancement, thereby improving the sensitivity, stability, and reproducibility of the substrate.

(2) Spectral analysis and signal processing are complex. The Raman spectral peaks of multiple harmful substances overlap. When detected by the direct method, it relies on multivariate statistical analysis (such as PCA) or imaging techniques to assist in analysis, increasing the difficulty of data processing. Although the indirect method enhances the sensitivity through Raman signal molecules (such as MBA, DTNB), excessive labeled molecules may generate background noise, affecting the detection of low-concentration substances. The development of multi-technology combination or multi-modal sensing technology is conducive to improving the accuracy and reliability of detection [162,163]. Furthermore, integrating artificial intelligence algorithms will also be beneficial for the analysis of spectral information and will enhance the accuracy [164].

(3) The on-site high-throughput detection capacity is insufficient, and there may be problems such as multiple signal interferences. At present, most SERS platforms can only simultaneously detect two to four substances, making it difficult to meet the demand for the coexistence of multiple harmful substances in actual samples. In addition, during multi-channel detection, there may be cross-interference in the signals of different detection areas. More efficient sample pretreatment techniques, such as accelerated solvent extraction and microwave-assisted extraction, are adopted, combined with magnetic nanoparticles for separation and enrichment, to reduce the interference of the sample matrix and improve the detection sensitivity [165]. By using portable devices and combining them with LFIA and microfluidic chip technology to develop array channels, it is conducive to achieving real-time and on-site detection of samples [166].

## 5. Conclusions

SERS-based simultaneous detection technology has garnered significant attention in the field of food contaminant detection due to its unique capabilities and has led to a range of successful outcomes. This article reviews the research progress in simultaneously detecting various harmful pollutants in food using SERS technology in the past five years. The main strategies can be categorized into two types: label-free detection and labeled detection. Label-free detection mainly obtains Raman characteristic peaks from multiple target substances and conducts qualitative/quantitative analysis based on each characteristic peak. In addition, the introduction of algorithms such as chemometrics usually improves the accuracy of this method. Raman reporter molecules are of great significance in label detection because they, as a source of signals, determine the effectiveness of sensing strategies. Labeled detection, on the other hand, can be further divided into spatial separation detection and SERS signal encoding. Spatial separation detection is usually carried out by collecting the signals of the corresponding Raman reporter molecules at different positions of the same device (such as flow immunochromatography test strips). The signal changes at different positions correspond to the concentration changes of different target substances. SERS signal encoding usually involves collecting the signals of probe molecules at the same location. Each probe molecule presents different Raman characteristic peaks, and the concentration of the corresponding target substance can be obtained based on these different Raman peaks. Although simultaneous SERS-based detection methods have made notable progress in food contaminant detection, challenges remain in practical applications. First, the stability and reproducibility of SERS substrates, particularly nano-sol–gel substrates, require improvement. In these substrates, nanoparticle aggregation disorder can lead to poor signal repeatability. Second, some food contaminants generate weak SERS signals, and food matrices may cause signal interference. While chemometrics and other algorithms can aid in data analysis, they also introduce additional complexity. Finally, this technology lacks high-throughput detection capabilities in real-world settings. To overcome these limitations, future advancements should focus on developing intelligent SERS substrates and refining preparation processes to enhance the uniformity and stability of nanoparticle hotspots. These intelligent substrates may achieve integration of “detection-cleaning (processing)-recycling”. Moreover, the development of multimodal sensing technologies and artificial intelligence algorithms can improve spectral information extraction and detection accuracy. Lastly, integrating LFIA, microfluidic chip technologies, and portable Raman spectrometers to create array channels will facilitate on-site detection of multiple samples.

## Data Availability

No new data were created or analyzed in this study. Data sharing is not applicable to this article.

## Abbreviations

The following abbreviations are used in this manuscript:

SERS	Surface-enhanced Raman scattering
HPLC	High-performance liquid chromatography
TBZ	Thiabendazole
LOD	Limit of detection
TRM	Thiram
ZEN	Zearalenone
PRQ	Paraquat
TYR	Tyramine
RAC	Ractopamine
MBA	4-mercaptobenzoic acid
DTNB	5,5′-dithiobis-(2-nitrobenzoic acid)
NTP	4-nitrothiophenol
ATP	4-aminothiophenol
MPBA	4-mercaptophenylboronic acid
MPY	4-mercaptopyridine
MBN	4-(mercaptomethyl) benzonitrile
PB	Prussian blue
*P. aeruginosa*	*Pseudomonas aeruginosa*
*S. aureus*	*Staphylococcus aureus*
*S. epidermidis*	*Staphylococcus epidermidis*
*M. smegmatis*	*Mycobacterium smegmatis*
PCR	Polymerase chain reaction
MMC	7-mercapto-4-methylcoumarin
TFMBA	2,3,5,6-tetrafluoro-4-mercaptobenzoic acid
*E. coli*	*Escherichia coli*
*S. typhimurium*	*Salmonella typhimurium*
Cy3	Cyanine 3
ROX	Carboxy-X-rhodamine
AFB1	Aflatoxin B1
OTA	Ochratoxin A
LFIA	Lateral flow immunoassay
NBA	Nile blue A
DON	Deoxynivalenol
3D-Psi	3D porous silicon
FB1	Fumonisin B1
IMI	Imidacloprid
PYR	Pyraclostrobin
ACE	Acetamiprid
CBZ	Carbendazim
CHL	Chlorothalonil
OXY	Oxyfluorfen
ZIR	Ziram
TGA	Thioglycolic acid
TCP	Thiacloprid
OXA	Oxamyl
2-MCE	2-mercaptoethylamine
MG	Malachite green
CV	Crystal violet
ENR	Enrofloxacin
ENX	Enoxacin
CPX	Ciprofloxacin
CAP	Chloramphenicol
AM	Amoxicillin
TC	Tetracycline
OFX	Ofloxacin
NEO	Neomycin
LIN	Lincomycin
AF-C_3_N_4_	Oxygen-linked graphitic carbon nitride
CIL	Class-incremental learning
COF	Covalent organic framework
MNPs@ConA	Lectin-functionalized magnetic nanoparticle
Ag@Au	Silver-core gold-shell
AMP	Antimicrobial peptide
MNPs	Magnetic nanoparticles
PGNAs/Si	PAMAM-based gold nanoassemblies on silicon wafer
CES	Co-recognition, enrichment, and sensing
VFA	Vertical flow immunoassay
DQT	Diquat
PLS-DA	Partial least squares discriminant analysis
SVR	Support vector machine regression
AuNSs	Gold nanostars
PMMA	Poly(methyl methacrylate)
NFT	Nitrofurantoin
PP	Penicillin potassium
TCH	Tetracycline hydrochloride
LEV	Levofloxacin
PCA	Principal component analysis
CNN	Convolutional neural networks
NN-EN	Non-negative elastic network
TLC	Thin-layer chromatography
AuNPs	Gold nanoparticles
APC	Ampicillin
TMIP	Dual-template imprinted polymers
CLE	Clenbuterol
AgNPs	Silver nanoparticles
FDTD	Finite difference time domain
CARS-PLS	Competitive adaptive weighted sampling–partial least squares
DFT	Density functional theory
AuNR-MF	Au nanorod-incorporated melamine foam
SEB	Staphylococcal enterotoxin B
BoNT/A	Botulinum neurotoxin type A
HIS	Histamine
RGO/AgNPs	Reduced graphene oxide/silver nanoparticles
